# A Few Facts Concerning the Recent Prevalence of Winter Diarrhœa

**Published:** 1887-12

**Authors:** Shirley F. Murphy

**Affiliations:** Lecturer on Hygiene and Public Health, St. Mary’s Hospital


### A FEW FACTS CONCERNING THE RECENT PREVALENCE OF WINTER DIARRHŒA.

BY SHIRLEY F. MURPHY, LECTURER ON HYGIENE AND PUBLIC HEALTH, ST. MARY'S HOSPITAL.

I have thought that our society might well have before it a few facts which I have been able to glean concerning the recent prevalence of diarrhœa during the winter months. The subject was first referred to in a paragraph in a recent number of *The Lancet*, which also contained a letter from Mr. C. H. Taylor of the West London Hospital, stating that a diarrhœa was prevalent in Fulham, Chiswick and Hammersmith; and the *British Medical Journal* of the same date published a communication from Dr. Norman Kerr, telling the same story for the neighborhood of Regent's Park. Later, other corresopndents bore witness to the existence of epidemic diarrhœa in St. James', Bayswater, Marylebone, Clapham, Lewisham, Penge, Kingston-on-Thames, Kent, and even as far from London as Lancashire and Aberdeenshire. The reports of the symptoms from which the patients suffered fairly well agreed. In all cases there was diarrhœa, in the majority abdominal pain, vomiting, cramps in the limbs, great depression, and a symptom of particular interest—high temperature. In a few, the fact was noted that the patients also suffered from hæmorrhage from the bowels. In many instances, the sufferers were attacked in the night, and often in groups, suggesting community of cause. We may assume therefore, in the first instance, that the epidemic, wherever it manifested itself, was of the same nature, and that some of the symptoms to which it gave rise where those not usually met with in ordinary diarrhœa, and that, regarding it as diarrhœa, the disease occurred at an unusual period of the year. We are led, moreover, to conclude that, notwithstanding the area which its operations covered, it is in the highest degree probable that the cause in each locality was the same. And I may go further, and say that this cause was not related to any climatic condition; for, if we examine the dates of outbreaks in public institutions situated near to each other, and therefore subject to the same climatic conditions, we do not find that these dates exactly correspond; and, further, some institutions also in the neighborhood of those which were invaded escaped altogether. The cause must, nevertheless, be one capable of operating over large extents of country.

My own opportunities for learning some particulars of this illness, were practically limited to its behavior in a special hospital in Central London, which I first visited for that purpose on January 27th. I was informed by the senior house-surgeon, who had taken great interest in the matter, that half the inmates of the institution, about two months before, had been attacked suddenly with diarrhœa, the patients alone suffering and the staff altogether escaping. On January 25th, the incident repeated itself, nearly fifty persons on this occasion being attacked. The distribution of the disease in the hospital is of importance; for again, on the second occasion, the patients were, with trifling exceptions, the only sufferers. The patients are resident in wards situated on three floors; those on each floor taking their meals mostly together. On the first floor are beds for thirty women, on the second floor beds for forty men, and on the third floor twenty-six beds, twenty-one of which are occupied by men and the rest by children. Of the patients resident in the hospital at the time of my visit, I found that on the first floor there were attacked on January 24th (Monday), in the morning one, in the evening three; on the 25th (Tuesday), in the morning two, in the evening six; on the 26th (Wednesday), in the morning one. On the second floor there were attacked on January 24th (Monday), in the evening one; on the 25th (Tuesday), in the morning eighteen. On the third floor there were attacked on January 24th (Monday), in the evening five; on the 25th (Tuesday), in the morning six. Or, considering those on the different floors together—on Monday, January 24th, ten were attacked; on Tuesday, 25th, thirty-two; and on Wednesday, the 26th, one; the majority of the attacks occurring while the patients were in bed, at some period of the night.

The most striking circumstance in the outbreak is the almost complete immunity enjoyed by the staff. Of twenty-two persons engaged either in attendance upon the sick or in domestic duties, but two suffered from diarrhœa, and these were nurses, both of whom were attacked in the early morning of Tuesday, January 25th.

To enable the exact incidence of the disease upon the patients to be determined, and for the purposes of further inquiry, I ascertained the condition of health of those who had been discharged from the hospital for some time previously, and in this manner elicited information that no patient who left the hospital before January 24th was attacked. On Thursday, January 20th, three patients were discharged, and on Friday, the 21st, three; on Saturday, the 22d, six—none of whom suffered. On Sunday, the 23d, no patients were discharged; on Monday, the 24th, six patients were discharged, three of whom were attacked early the following morning at their own homes. Whatever cause, therefore, gave rise to diarrhœa probably came into operation after the patients left on January 22d—*i. e.*, after mid-day dinner on that day. Of patients newly admitted into the hospital, three admitted on January 20th were each attacked; of two admitted on the 21st, one was attacked; and of three admitted on the 22d, two were attacked. None were admitted on Sunday, January 23d. Five were admitted on January 24th, all of whom escaped. Two were admitted on January 25th, both of whom escaped. The cause of the outbreak, therefore, probably ceased to be operative before the patients were admitted on Monday, January 24th—*i. e.*, the cause was operative only sometime between mid-day on Saturday, January 22d, and mid-day on Monday, January 24th.

It becomes important, therefore, to ascertain the effect of this cause upon the patients *who were in the hospital at this particular time*—changes by admissions and discharges having taken place before my visit. There were sixty patients in the hospital between January 22d (Saturday), mid-day, and January 24th (Monday), mid-day, of whom forty-three were attacked with diarrhœa, either within the hospital or immediately after their return home, giving a percentage of 76·6. Of the staff I have already said there were twenty-two, of whom two were attacked, or 9 per cent. This difference is so great that I was compelled to regard the condition causing the diarrhœa as one with which the patients must be specially related; and this view was strengthened by the fact that the two affected members of the staff were nurses, who may likely enough have come into accidental relation with this condition.

At this period I was not aware that the disease was prevalent in other parts of England, and I was led to examine the incidence of attack upon patients taking milk, water, and other articles of food. Of those who drank unboiled water at their meals there were thirty-three, of whom twenty-five were attacked, or 75·7 per cent., being practically identical with the general average. Of those who drank no water except that which they had in their tea, and which had therefore been boiled, there were twenty-six, of whom twenty-one were attacked, or 80·7 per cent. Drinking water or abstaining from water did not, therefore, affect the probabilities of attack. Again, forty-four patients drank uncooked milk instead of beer, and of these thirty-four were attacked, or 77·2 per cent.—a proportion equal to the general average. Of fifteen patients who took no milk except in tea, thirteen were attacked, or 86.6 per cent. Drinking or abstaining from milk cannot, therefore, be said to have affected the probabilities of attack. Nevertheless, the large proportion of attacks among patients, and the immunity enjoyed by the staff, strongly indicated some article of food as responsible for the occurrence. The senior house surgeon was good enough to prepare for me a complete list of all the articles of food consumed in the hospital. I may at once eliminate from further suspicion the following: Milk, because the supply was common to staff and patients, because of the evidence already adduced, and also because the same farms supplied milk to another large hospital, which remained free from disease. Water, because it was consumed by staff and patients, and because of the evidence already adduced. The possibility that the local contamination of a cistern was responsible for the outbreak may be set aside by consideration of the fact that separate cisterns supplied the different floors. Tea and salt may be eliminated, because the supply was common to staff and patients. Meat and potatoes, because the supply was common to staff and patients. Beef-tea, because it was only consumed by a few patients. Beer, because those who did not drink beer were also attacked. Bread and sugar, because the same articles consumed by the patients were also supplied to the servants, none of whom were attacked. Rice, because no fresh supply had been received for a long time previously, and the children had been eating rice daily without injury. I may also refer to the fact that other institutions and other parts of the country were invaded, having different milk, water and meat supplies.

There is but one article of food I am unable to exculpate; and, to the possibility of this being the cause of a widespread outbreak, I would desire to direct the attention of the society. I refer to butter, which in the method of its distribution in the hospital in the first instance raises a presumption that it may have been concerned in producing this curious disease. Three qualities of butter were supplied to the institution—(*a*) that provided for the medical officers and matron, (*b*) that provided for the nurses and servants, and (*c*) that provided for the patients. The patients' butter is received on every week-day, at 10 A. M., and is used for tea and supper the same day, and for breakfast the following day, on Saturdays the supply being increased to last until breakfast on the following Monday morning. If we assume that the butter received for the patients on Saturday, January 22d, possessed purgative properties, there is ample explanation of the illness of all the patients who suffered from diarrhœa. That all were not attacked at the same time, and that some escaped, may be due to some unequal distribution of the active principle throughout the butter, or perhaps to some difference in the quantity consumed. There remain the attacks of two nurses, who, it may be mentioned, suffered less severely than the patients from this malady. I have no other explanation to offer on this point than that they must have eaten some portion of the bread and butter not required for the patients. Those who are intimate with hospital life will know that this is no unusual event. Thus far as to this particular hospital. The difficulty which stands in the way of the acceptance of this theory is the fact that other institutions — many persons not living in institutions, and other parts of the country have also shared in the prevalence of diarrhœa.

English butter is made upon the farm on which the milk is produced, and the few pounds which each farm is able to contribute could not be sufficiently widely distributed to give rise to an occurrence such as that we have under consideration. But the butter which I have been led to suspect was that provided for the patients, and low-priced butters come largely from abroad. In other countries butter is not always manufactured on the farms on which the milk is produced; but large numbers of farms send their milk to central depots, where, I am told, the milk of hundreds of farms is mixed together, made into butter, and coloring matter added, which always gives to it a uniform color and flavor. This is sent over in enormous quantities to the English middle-man, who distributes it to local vendors, from whom it reaches the consumers. In this manner any capacity which the milk from any farm might possess to cause diarrhœa would, if due to an organism, have the possibility of being multiplied by admixture of this milk with other milk, and thus its effects might be felt in many places. Or, perhaps, the method of treatment of the butter in some central depot might be concerned in conferring upon it this power, or possibly by the development of some chemical poison it might have acquired this special property. The symptoms of the sufferers, indeed, remind us of the observations of Dr. Victor C. Vaughan, of Michigan, concerning the effects of a ptomaine he found in cream. It is, under any circumstance, conceivable that butter which possesses this property might be distributed through different channels over a large area of country, and thus induce diarrhœa in one district or institution at one time and in another at the same or at a different time.

I have not had the opportunity of extending my inquiries sufficiently to enable me to thoroughly sift the evidence that could be obtained outside the institution with which I was concerned. I therefore prefer to leave the subject where it is; but I may mention incidentally that one large hospital, which also suffered heavily, received its butter from the same source as that which came under my own observation, and that another suffering very partially required the patients to supply their own butter—thus giving opportunity for it to be procured from many sources, of which perhaps one only was identical with that under suspicion. This difference in the method of supply may perhaps account for the smallness of the number of persons affected in some institutions. I do not wish now to do more than urge—1st, that the nature of the epidemic of which we have recently had experience points to an article of food as the cause; 2d, that the facts learned at one institution, where they were carefully observed, point especially to butter; and, 3d, that any objection to this theory, which is based on the method of distribution of butter made on English farms, falls to the ground when the distribution of foreign and of artificial butters is considered. In conclusion, I would desire very cordially to thank Mr. E. Treacher Collins, whose active assistance has enabled me to ascertain the facts upon which this account is based, and to whose accurate observation I am very greatly indebted—*London Lancet.*

